# Supplementary material for: SKIOME Project: a curated collection of skin microbiome datasets enriched with study-related metadata
Source: Database (Oxford). 2022 May 16;2022:baac033. doi: 10.1093/database/baac033 (PMC9216470; doi:10.1093/database/baac033)
Supplement: baac033_Supp [file baac033_supp.zip › Agostinetto_supplementary_file_description.docx]

**Supplementary File**

Here we provide a list with the description of Supplementary materials (the copy of our Github repository, currently private) accompanied by the link to access to them.

Link:

https://drive.google.com/drive/folders/1HY8CDaqAIWBEGtNuawY1v4LUeN95tYy5?usp=sharing

File description:

- Supplementary_file_1.csv

Comprehensive list of the manually curated metadata with description

- README.md

README of our Github repository

- skiome_workflow.png

Figure representing the metadata retrieval workflow

- SKIOME_notebook.ipynb

Complete pipeline as Jupyter notebook for dataset and metadata retrieval

- SKIOME_pipeline.Rmd

Complete pipeline in Rmd format for dataset and metadata retrieval

- Human_Skin_Datasets_Manual_Search.csv

Result of the manual search for the datasets and metadata

- skiome_dataframes.zip
  Compress file containing the three dataframes obtained from our work
